# Prenatal maternal sleep and maternal-infant attachment: a systematic review

**DOI:** 10.3389/frsle.2025.1626006

**Published:** 2025-08-11

**Authors:** A. J. Crandall, Mia Ayala Garcia, Neriah Jones, Pearl Ayiku, Claudia Lugo-Candelas

**Affiliations:** ^1^Child and Adolescent Psychiatry, Columbia University Irving Medical Center, New York, NY, United States; ^2^Child and Adolescent Psychiatry, New York State Psychiatric Institute, New York, NY, United States

**Keywords:** prenatal sleep, maternal-infant attachment, pregnancy, child development, maternal health

## Abstract

**Background:**

Maternal-infant attachment is essential for children's socioemotional and cognitive development. Secure attachment supports emotional regulation, while insecure attachment is linked to adverse mental health outcomes. While prenatal stress and depression are known predictors of attachment, the impact of prenatal maternal sleep is underexplored, despite poor sleep affecting 75% of pregnant individuals by the third trimester. Given the overlap between sleep disturbances, stress, and depression, disrupted sleep may contribute to impaired infant attachment. This systematic review evaluates evidence linking prenatal sleep and maternal-infant attachment.

**Methods:**

We searched PUBMED, Web of Science, EMBASE, PsycINFO, and SCOPUS for studies published from 2000 to 2024. Studies were included if they quantitatively examined associations between prenatal maternal sleep and attachment assessed up to 5 years postpartum. Two reviewers independently screened studies and extracted data. Quality was appraised using the NHLBI tool. Of 2,539 articles, 1,263 unique studies were screened, and only two met the criteria.

**Results:**

Both studies relied on maternal self-reports. One found prenatal snoring predicted weaker bonding at 6–9 weeks postpartum; another found no direct effects of sleep duration but identified an indirect link to maternal depression. Given limited and mixed findings, further research using objective and subjective sleep measures is needed.

## Introduction

Maternal attachment, or the emotional bond between a mother and their infant, is fundamental to the child's development and overall wellbeing (Karakas and Dagli, 2019; Poeira and Zangão, 2022). Secure maternal-infant attachment is associated with positive outcomes such as better emotional regulation, enhanced social skills, and improved cognitive development skills (Sagone et al., 2023). By definition, a securely attached infant has developed a trusting relationship and bond with their caregiver. This sense of security enables the infant to confidently explore their environment trusting that they have the support and comfort of their caregiver (Karakas and Dagli, 2019). Conversely, insecure attachment is linked to negative consequences such as mental health problems like depression and anxiety, behavioral issues, and difficulty forming relationships (Lee and Hankin, 2009). Insecure attachment in infants can develop when caregivers are unresponsive, or inconsistent, and infants are left uncertain whether their needs will be met. Oftentimes, infants with an insecure attachment may display heightened responses to stress and difficulty calming down (Karakas and Dagli, 2019). Understanding the determinants of maternal-infant attachment is crucial for developing effective interventions aimed at preventing attachment-related issues between a mother and her infant.

Previous research has extensively investigated how prenatal stress and depression influence attachment. Findings consistently indicate that elevated prenatal stress and depression are linked to insecure attachment, even when controlling for postnatal depression and other confounding variables (Bind et al., 2021; Monk et al., 2012). Although studies underscore the importance of prenatal maternal mental health, a critical aspect of prenatal health, sleep health, has not received nearly the same attention. This is of concern, as prenatal sleep has been associated with maternal and infant outcomes (Lugo-Candelas et al., 2023; Pauley et al., 2020), and poor sleep in pregnancy is highly prevalent, with up to three out of four pregnant persons endorsing poor sleep in the third trimester (Takelle et al., 2022).

Pregnant persons often experience worse sleep as the pregnancy progresses due to changes in hormones, respiration, cardiovascular function, fetal movement, and the frequent need to urinate throughout the night caused by a growing fetus (Lu et al., 2021). As sleep disturbances are common in the context of prenatal stress and depression, it raises the question of whether disrupted sleep could be an underlying factor contributing to the issues with attachment seen in studies of prenatal stress and depression. Sleep problems during pregnancy are associated with negative consequences for both the mother and child (Peltonen et al., 2023), including prenatal maternal depression and postnatal sleep issues, which can impact mothers' ability to bond with their infants (Peltonen et al., 2023). Of note, prenatal sleep disturbances are also associated with sleep problems and socioemotional difficulties in offspring (Lugo-Candelas et al., 2023) raising the possibility that difficulties with attachment may underlie associations between poor maternal sleep and poor offspring outcomes (Peltonen et al., 2023).

In sum, prenatal sleep is a crucial, yet often unexplored, component of prenatal maternal health that could significantly impact maternal-infant attachment. Considering prenatal sleep is a modifiable risk factor, understanding the role of prenatal sleep will offer valuable insights into attachment formation. This systematic review seeks to understand the relationship between prenatal sleep and maternal-infant attachment.

## Methods

### Inclusion and exclusion criteria

We included peer-reviewed quantitative studies that examined the association between prenatal maternal sleep and maternal-child attachment. Associations between prenatal maternal sleep and maternal-child attachment did not need to be the main objective of the study. Studies must have examined sleep during the prenatal period and included a measure of maternal-child attachment assessed between infancy and childhood, up to 5 years of age. Studies were excluded if they only reported on postnatal sleep, did not differentiate between pre- and post-natal sleep, were pre-clinical studies, studies of sleep disorders other than insomnia or parasomnias (e.g., sleep apnea, REM disorders), or did not assess postnatal attachment. Clinical trials involving ongoing treatment for sleep disorders that did not have baseline data were also excluded. Sleep assessment methods could include interviews, self-reports, polysomnography, actigraphy, and other established approaches. Attachment could be assessed via interviews, self-report surveys, video assessments, and other established approaches. Only articles in English and Spanish published between the years of 2000–2024 were included. There were no restrictions on the study setting, context, or age of mothers.

### Search strategy

This systematic review was conducted according to the Preferred Reporting Items for Systematic Reviews and Meta-Analyses guidelines (Page et al., 2021). The search strategy, inclusion criteria, and data extraction fields were established by the research team and registered prior to initiating the review (PROSPERO CRD42024518450). PUBMED, Web of Science, EMBASE, PsycINFO, and SCOPUS were searched using a search strategy that included terms related to (1) prenatal maternal sleep and (2) maternal-child attachment. See Supplementary data for the search strategy. The search was conducted from May 16 to June 7, 2024.

**Figure 1:**
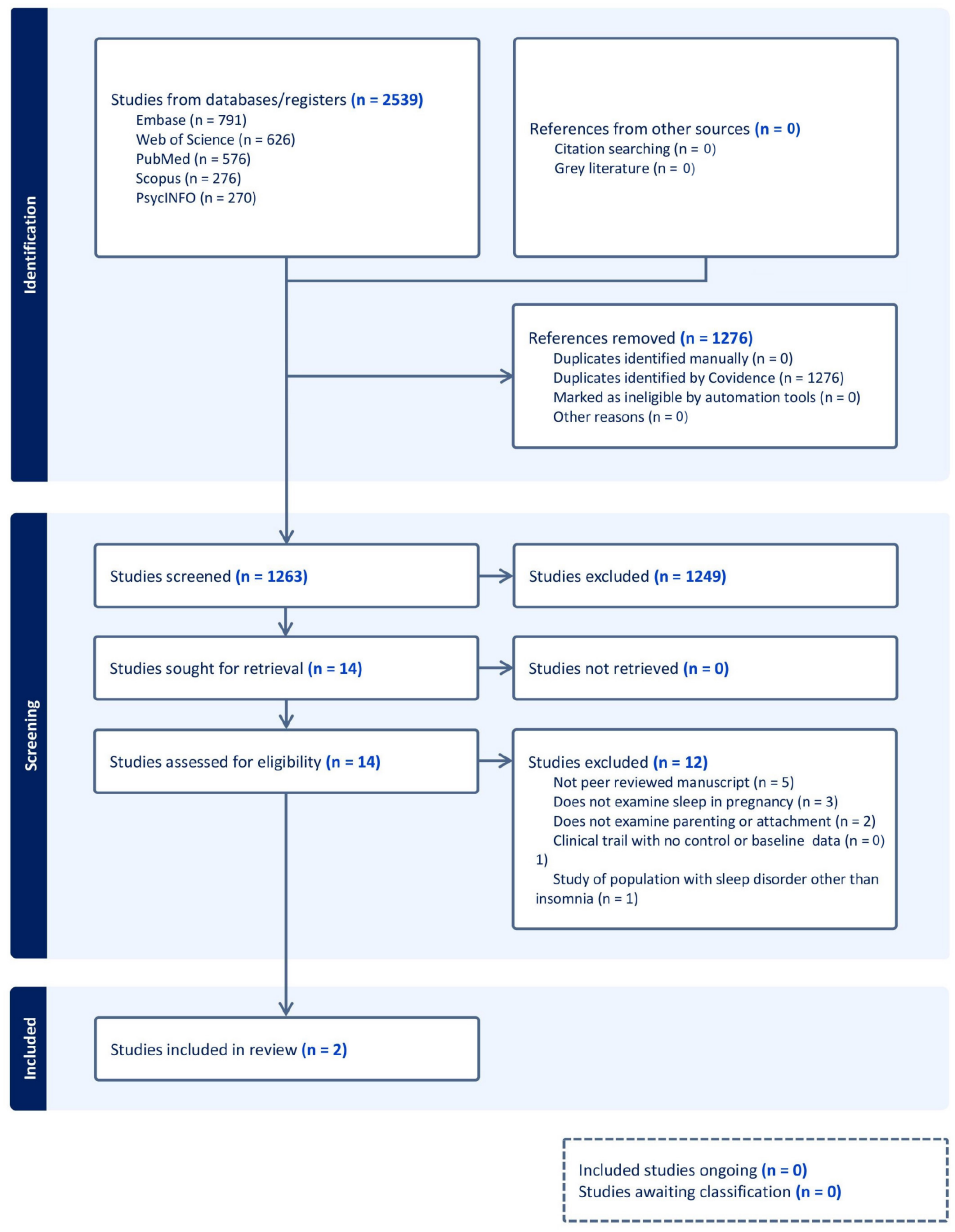
PRISMA flowchart.

The search yielded 2,539 results (1,263 unique studies after duplicates were removed). After initial title and abstract screening, 14 studies underwent full text review. During full text review, 12 studies were excluded, with the main reasons for exclusion being: not being a peer reviewed manuscript (*n* = 5), not examining sleep during pregnancy (*n* = 3), not examining parenting or attachment (*n* = 2), being a clinical trial with no baseline data (*n* = 1) and a study of a population with sleep disorder other than insomnia (*n* = 1). After meeting the inclusion/exclusion criteria, two studies underwent extraction (Figure 1).

For screening, review, and extraction, reviewers used covidence to organize and review all studies. At both the title and abstract level as well as the full text review, two reviewers independently assessed each article. If there were any discrepancies, a third reviewer was brought in to make a final determination.

### Data extraction and synthesis and quality appraisal

The extracted data included publication year, study aims, sample description (such as sample size, country, prenatal age, socioeconomic status, race/ethnicity), study design, inclusion and exclusion criteria, and type and timing of sleep and attachment measures used and covariates included in analyses. All extracted data were compiled into summary tables (see Supplementary Tables S1, S2).

The quality of the included studies was evaluated using the National Heart, Lung, and Blood Institute (NHLBI) Quality Assessment Tool for Observational Cohort and Cross-sectional Studies (NHLBI, 2013). This tool assesses the risk of bias across 14 criteria, focusing on key concepts for the internal validity of the studies.

## Results

### Description of the included studies

Two studies met eligibility criteria and were included in this systematic review. Included studies were published in the years 2016 and 2022 and conducted in the United States. The mean age of pregnant participants was 29 years old. One study conducted a postnatal attachment assessment between 6 and 9 weeks post-partum while the other assessed attachment at 30 months of age. Whereas one study was mostly comprised of participants who self-identified as White (88%), the other study was composed of a more diverse sample (only 58.2% identified as White). Only one study reported on the education attainment of their sample, and it showed a highly educated sample where 87% of pregnant participants reported completing at least some college. One study reported that 18.5% of their sample was classified as living in poverty while the other sample reported that most of their sample was middle or upper SES. The two studies assessed prenatal sleep via maternal self-report, no objective sleep measures were obtained (Table 1).

**Table 1:** Characteristics of the included studies and results.

**References**	**Sample size, maternal age (*M* ±SD years), race and ethnicity (%)**	**Setting**	**Socioeconomic status of sample**	**Sleep variable assessed and GA at assessment**	**Attachment variable assessed and child age at assessment**	**Summary of findings**
Kalmbach et al., 2021	66 dyads. Mean maternal age = 29.42. Sample was: White: 58.2% Black: 23.9% Asian: 4.5% Middle Eastern or Arabic: 3.0% Hispanic or Latino: 6.0% Multiracial: 4.5%	United States	18.5% classified as living in poverty (operationalized as < $20,000 annual household income). Education NR	Insomnia severity, pre-sleep arousal, nocturnal perinatal-focused rumination, daytime sleepiness, and snoring measured by the Insomnia Severity Index (ISI) were assessed starting baseline assessment (gestational weeks 25 and 30) and weekly across the remainder of pregnancy	Postpartum Bonding Questionnaire (PBQ) parent report form completed weekly across the first two postpartum months (newborn—~8 weeks old)	Pregnant persons who reported snoring during pregnancy had significantly poorer mother-to-infant bonding after childbirth Miscarriage, maternal age, race, poverty status, BMI, prenatal insomnia, prenatal maternal rumination, prenatal depression, nocturnal cognitive arousal during pregnancy and daytime sleepiness were not associated to bonding
Newland et al., 2016	132 dyads. Mean maternal age = 29 Sample was: 88% White non-Hispanic, 12% minority racial status	United States	5.7% less than a high-school education, 9.8% high school graduates, 28.9% completed some college, 36.7% college graduates, 17.9% had graduate training	Mean hours of sleep prenatally (including nighttime and naps)	Attachment Q-Set home observational measure at 30 months of age	Prenatal maternal sleep duration was not significantly correlated with attachment security at 30 months. The emergence of a secure mother-child relationship was predicted, in part, by infant sleep through its effect on maternal depression, but only for mothers and children with poorly matched sleep patterns

### Description of findings

(Kalmbach et al. 2021) completed a cohort study with participants recruited from a larger parent study on perinatal health from a multi-hospital health system. Eligible participants were then assessed for prenatal sleep at 25–30 weeks gestational age through one item about snoring, an insomnia severity index, one item assessing perinatal rumination, and one item assessing whether pregnant participants had trouble staying awake while driving, eating meals, or engaging in social activity. Offspring attachment was assessed by parental report through the Postpartum Bonding Questionnaire (PBQ) at 6–9 weeks post-partum. Pregnant persons who reported greater snoring and weak maternal fetal attachment during pregnancy self-reported poorer maternal-infant bonding post-partum, even when controlling for miscarriage, race, poverty, maternal-fetal attachment, prenatal maternal obesity, depression, and rumination, and time of assessment. Prenatal daytime sleepiness and insomnia were also included in the models but did not predict postnatal attachment. A methodological strength includes the relative diversity of the sample. Weaknesses in the study design include exclusive use of self-report measures and whether the weekly assessments can capture temporal effects of sleep in the postnatal period, as stated by the authors.

On the other hand, (Newland et al. 2016) assessed prenatal sleep in a cohort study recruited from a hospital in northeastern United States. During the third trimester of pregnancy, with an instrument (name not provided) that included general questions about sleeping habits and mean hours of sleep, which included overnight sleep and naps. Offspring attachment was assessed through the 90-item Attachment Q-Set at 30 months of age (Vaughn and Waters, 1990). Prenatal nighttime sleep duration was not significantly associated with maternal-child attachment while controlling for socioeconomic status and prenatal maternal depressive symptoms. However, prenatal maternal sleep duration was not completely unrelated to postnatal attachment, as interactions between prenatal maternal and infant sleep duration at 8 months indirectly affected maternal-child attachment security at 30 months, via increasing maternal depressive symptoms. Weaknesses in the study design include a non-racially and economically diverse sample, high proportions of missing sleep data and use of self-report sleep measures across the perinatal period. A strength to highlight from this study is the longitudinal design, with the study participation spanning from prenatal to 30 months post birth.

## Discussion

The present study aimed to summarize the available evidence on the association between prenatal maternal sleep health and maternal-infant/child attachment. Studies found that prenatal snoring negatively impacted maternal-child attachment. While other prenatal sleep metrics, such as sleep duration, rumination, insomnia, and daytime sleepiness, were not associated with difficulties in maternal-infant attachment, these were found to have downstream effects on attachment via increasing postnatal depression and interacting with the infant's sleep duration. Whereas this review is inconclusive given the limited number of studies available we discuss potential factors influencing mixed results and the gaps in the literature future studies should aim to address.

Given that only two studies met our inclusion criteria, it is important to acknowledge potential methodological and conceptual barriers that may limit investigation in this research area. One key challenge is the logistical and financial burden of collecting longitudinal data from the early stages of pregnancy. Many existing studies rely on cross-sectional data, which cannot establish temporal precedence or causality between maternal sleep problems and mother-infant bonding (Kalmbach et al., 2021). Only a very limited number of prospective longitudinal studies exist, which are crucial for understanding how prenatal sleep disturbances may predict postnatal attachment outcomes. Another limitation is that both included studies relied solely on self-reported sleep data, which are subject to recall bias and may not accurately capture sleep quality or disturbances. Future research would benefit from incorporating objective sleep measures, such as actigraphy or polysomnography, to improve data reliability. Additionally, difficulty accessing these tools in obstetric cohorts and conceptual “siloing” between fields may also play a role, as sleep and attachment researchers often operate in parallel but disconnected domains, publishing in different spaces and rarely collaborating. These factors may partly explain why so few studies exist. A more integrative and interdisciplinary research approach, along with investment in prospective, multimethod study designs, may help address this critical gap in the literature.

(Kalmbach et al. 2021) found prenatal snoring, and not insomnia, to be related to mother-child attachment at 6–9 weeks postpartum. This finding, although requiring replication and further examination of the underlying mechanisms, may suggest that pregnant persons with breathing difficulties may be at risk for disruptions in postnatal attachment. Of note, prior studies have documented associations between snoring during pregnancy and risk for cardiometabolic complications (Laposky and Pemberton, 2020), coronary artery disease and depressive disorder (Bhattacharyya, 2015), possibly suggesting that pregnant persons with a more complicated perinatal health profile could be a population at greater risk for postnatal attachment disruptions. In fact, prenatal snoring has been associated with poorer maternal health outcomes during pregnancy, including gestational hypertension, preeclampsia (O'Brien et al., 2012) increased fetal distress (Wang et al., 2024), complications with delivery (including increased cesarean section rates) (Okun and O'Brien, 2018), and low APGAR scores after birth delivery (Robertson et al., 2022). It is thus possible that these dyads are at a higher risk for health issues (O'Brien et al., 2013), which may in turn impact post-natal attachment. Few studies of pregnancy complications and postnatal bonding or attachment exist, yet suggest increased pregnancy complications may impact postnatal bonding via depression (Solomonova et al., 2020; Yang et al., 2023), but more research is needed. As we previously note, a key methodological limitation is that both included studies relied solely on self-reported sleep measures. Self-report often underestimates symptoms such as snoring or apneas and cannot capture sleep architecture. Of note, self-report is not a comprehensive method to measure snoring and the existence of related complications like sleep apnea. As individuals may not be aware of the severity or extent of their snoring during sleep, snoring could be under-reported and thus the strength of this association may be underestimated in this study (Kim et al., 2024). Studies may consider the use of new technological advances to measure snoring via remote sensors and other technologies suited for large epidemiological studies.

On the other hand, (Newland et al. 2016) found that although prenatal sleep duration did not directly impact postnatal attachment, interactions between prenatal maternal sleep duration and infant sleep at 8 months indirectly affected attachment security via maternal depressive symptoms. Because findings were only present in dyads with poorly matched sleep patterns (i.e., mothers that report sleeping for long periods of time during pregnancy with infants that have unpredictable sleep patterns), it was hypothesized that mothers who reported longer sleep duration during pregnancy might have greater difficulty adapting to an infant with higher sleep difficulties. This pathway has not been widely researched but could demonstrate the importance of considering prenatal maternal sleep profiles and “mismatches” between maternal and infant sleep habits when determining postnatal depression risk and need for attachment support. This pathway would also suggest that exclusively considering postnatal sleep health (infant and maternal) when seeking to prevent attachment problems and postpartum depression may be leaving out an important part of the equation that preceded birth, prenatal sleep duration. Furthermore, prenatal sleep disturbances increase risk for children's own sleep problems (Lugo-Candelas et al., 2023), highlighting the need to deploy interventions to improve sleep prior to birth. However, more research is needed to better understand these longitudinal associations.

Interpreting the existing findings is further complicated by heterogeneity in attachment assessment. The two studies employed different tools, the Postpartum Bonding Questionnaire (PBQ) at 6–9 weeks and the Attachment Q-Set at 30 months, capturing distinct developmental windows and methodologies, such as self-report vs. structured observation. Such variation limits comparability and may obscure temporal dynamics, as previously discussed. Harmonizing measurement approaches and adding repeated assessments will be crucial for synthesis in future meta-analyses.

The present review is limited due to only two studies of this nature existing. Studies that include comprehensive and objective measures of maternal and infant sleep are needed. Further, studies differed in their measurement of attachment (self-report vs. observation) and the timing of assessment. The study that documented direct associations between prenatal sleep and postnatal maternal-infant attachment assessed attachment early, at 6–9 weeks post birth, compared to 30 weeks in the second study, suggesting that either the impact of prenatal sleep on postnatal attachment is short lived and does not persist to toddlerhood, or that there are postnatal factors that buffer the impact of poor prenatal sleep on long-term attachment disruptions that could be leveraged in interventions. Further studies are required to better understand the impact of prenatal sleep across infancy through toddler and childhood. Future research should explore plausible biological and psychological mechanisms, including dysregulation of the hypothalamic-pituitary-adrenal (HPA) axis, altered oxytocin signaling, inflammatory pathways, and heightened maternal stress reactivity, that may link disrupted prenatal sleep to later caregiving sensitivity and attachment security (O'Donnell et al., 2013).

In sum, this review is inconclusive in documenting direct associations between prenatal sleep and maternal-infant/child attachment. Further research that determines the potential for prenatal sleep as an intervention target in the prevention of difficulties with attachment is needed.
