# Supplementary material for: Prenatal maternal sleep and maternal-infant attachment: a systematic review
Source: Front Sleep. 2025 Aug 11;4:1626006. doi: 10.3389/frsle.2025.1626006 (PMC12713894; doi:10.3389/frsle.2025.1626006)
Supplement: Supplementary file 1 [file Data_Sheet_1.docx]

**Supplemental Material**

**Search strategy.**

(attachment OR parenting) AND (sleep OR circadian) AND (infant OR child OR early childhood OR toddler

OR baby OR preschool OR childhood) AND (prenatal OR perinatal OR antenatal OR postnatal OR

peripartum OR postpartum).

**Inclusion and exclusion criteria.**

**Inclusion**

- Human subjects: pregnant or previously pregnant persons.
- Studies must specifically address sleep during the prenatal period.
- Sleep assessment methods may include interviews, self-reports, polysomnography, actigraphy, and other established approaches.
- Studies must include a measure of mother-child attachment assessed between infancy and childhood (before age 5).
- Attachment measures can be interviews, self-report surveys, video assessments and other established approaches.
- Peer-reviewed investigations that document potential correlations between prenatal maternal sleep and mother-infant attachment will be included.
- From 2000 until now.
- Studies must be in English or Spanish.
- Any study setting, context, or the age of pregnant participants.

**Exclusion**

- Studies of persons with a diagnosed sleep disorder other than insomnia and parasomnias.
- Studies of persons undergoing pharmacological and psychological treatments for sleep disorders.
- Any study that combines pre- and postnatal sleep assessments and we are unable to distinguish effect of prenatal sleep.
- Studies that do not measure attachment in children before or on age 5.
- Clinical trials and studies involving individuals undergoing sleep treatment that lack baseline data. In the case of clinical trials, only baseline information will be considered.
- Studies exclusively focusing on sleep disorders such as sleep apnea and narcolepsy, or those targeting populations with sleep disorders other than insomnia and parasomnias
- Animal studies.

The search was conducted in November 2023. We did not restrict the search by country or location.

Table 1. Characteristics of the included studies and results

| **Author and year** | **Sample size, maternal age (M**±**SD years), race and ethnicity (%)** | **Setting** | **Socioeconomic status of sample** | **Sleep variable assessed and GA at assessment** | **Attachment variable assessed and child age at assessment** | **Summary of findings** |
| --- | --- | --- | --- | --- | --- | --- |
| *Kalmbach et al., 2022* | 66 dyads. Mean maternal age = 29.42. Sample was: White: 58.2% Black: 23.9% Asian: 4.5% Middle Eastern or Arabic: 3.0% Hispanic or Latino: 6.0% Multiracial: 4.5% | United States | 18.5% classified as living in poverty (operationalized as < $20,000 annual household income). Education NR. | Insomnia severity, pre-sleep arousal, nocturnal perinatal-focused rumination, daytime sleepiness, and snoring measured by the Insomnia Severity Index (ISI) were assessed starting baseline assessment (gestational weeks 25 and 30) and weekly across the remainder of pregnancy. | Postpartum Bonding Questionnaire (PBQ) parent report form completed weekly across the first two postpartum months (newborn - ~8 weeks old). | Pregnant persons who reported snoring during pregnancy had significantly poorer mother-to-infant bonding after childbirth.  Miscarriage, maternal age, race, poverty status, BMI, prenatal insomnia, prenatal maternal rumination, prenatal depression, nocturnal cognitive arousal during pregnancy and daytime sleepiness were not associated to bonding. |
| *Newland et al., 2012* | 132 dyads. Mean maternal age = 29  Sample was: 88% White non-Hispanic, 12% minority racial status | United States | 5.7% less than a high-school education, 9.8% high school graduates, 28.9% completed some college, 36.7% college graduates, 17.9% had graduate training | Mean hours of sleep prenatally (including nighttime and naps). | Attachment Q-Set home observational measure at 30 months of age | Prenatal maternal sleep duration was not significantly correlated with attachment security at 30 months. The emergence of a secure mother-child relationship was predicted, in part, by infant sleep through its effect on maternal depression, but only for mothers and children with poorly matched sleep patterns. |

Table S1. Additional study details

| **Author and year** | **Maternal age** | **Covariates** | **Inclusion Criteria** | **Exclusion Criteria** | **Estimates, Effect sizes, P-values (Measures of association or group differences)** | **Comorbid conditions assessed** |
| --- | --- | --- | --- | --- | --- | --- |
| *Kalmbach et al., 2022*(1) | 20-39 years average = 29.42 | Miscarriage, age, race, poverty, BMI, maternal fetal attachment,  Prenatal ISI, Prenatal EPDS, Prenatal PSASC, Prenatal PFR, daytime sleepiness | 25 - 30 gestational weeks at screening | Conditions constituting a high risk pregnancy per self-report at baseline screening (e.g., pre-eclampsia diagnosis, age > 40 years; hypertension and diabetes were not exclusionary), being monitored by the maternal-fetal medicine team for high risk pregnancy, multiple pregnancy, use of prescription or over-the-counter sleep aids or any other medications or alcohol or recreational drug use at screening, rotating and/or night shift work, epilepsy or seizures, bipolar disorder, diagnosis of a sleep disorder that is untreated (other than insomnia), and severe depression. | Snoring: *b* = 8.30, 95/%CI = 2.89, 13.70, *p* = 0.003 | Maternal depressive symptoms, BMI |
| *Newland et al., 2012*(2) | 17-45 years  average = 29 | Socioeconomic status, prenatal maternal depressive symptoms, gender | Pregnant persons, half had a mother with a history of maternal depression | Not reported. | In correlation table *r* = -0.10, ns, in SEM analysis *b* = 0.04, *p* = ns | Maternal depression, infant sleep |

**Notes.** BMI = body mass index, ISI = insomnia severity index, EPDS = Edinburgh postnatal depression Scale, PSASC = pre-sleep Arousal Scale, PFR = perinatal-focused rumination, NS = not signifigant

Table S2. Study quality assessment.

| **Criteria / Author and Year** | **Kalmbach 2022** | **Newland 2012** |
| --- | --- | --- |
| 1. Was the research question or objective in this paper clearly stated? | Yes | Yes |
| 2. Was the study population clearly specified and defined? | No | Yes |
| 3. Was the participation rate of eligible persons at least 50%? | No | NR |
| 4. Were inclusion and exclusion criteria for being in the study prespecified and applied uniformly to all participants? | Yes | Yes |
| 5. Was a sample size justification, power description, or variance and effect estimates provided? | No | No |
| 6. For the analyses in this paper, were the exposure(s) of interest measured prior to the outcome(s) being measured? | Yes | Yes |
| 7. Was the timeframe sufficient so that one could reasonably expect to see an association between exposure and outcome if it existed? | Yes | Yes |
| 8. For exposures that can vary in amount or level, did the study examine different levels of the exposure as related to the outcome? | Yes | Yes |
| 9. Were the exposure measures clearly defined, valid, reliable, and implemented consistently across all study participants? | Yes | No |
| 10. Was the exposure(s) assessed more than once over time? | Yes | No |
| 11. Were the outcome measures clearly defined, valid, reliable, and implemented consistently across all study participants? | Yes | Yes |
| 12. Were the outcome assessors blinded to the exposure status of participants? | NA | NR |
| 13. Was loss to follow-up after baseline 20% or less? | Yes | No |
| 14. Were key potential confounding variables measured and adjusted statistically for their impact on the relationship between exposure(s) and outcome(s)? | Yes | No |

**Notes**. NA = Not Applicable; NR = Not Reported
